# Retrospective study: The diagnostic accuracy of conventional forceps biopsy of gastric epithelial compared to endoscopic submucosal dissection (STROBE compliant)

**DOI:** 10.1097/MD.0000000000004353

**Published:** 2016-07-29

**Authors:** Chao Lu, Xueyou Lv, Yiming Lin, Dejian Li, Lihua Chen, Feng Ji, Youming Li, Chaohui Yu

**Affiliations:** Department of Gastroenterology, The First Affiliated Hospital, College of Medicine, Zhejiang University, Hangzhou, China.

**Keywords:** conventional forceps biopsy, concordance, endoscopic submucosal dissection

## Abstract

Conventional forceps biopsy (CFB) is the most popular way to screen for gastric epithelial neoplasia (GEN) and adenocarcinoma of gastric epithelium. The aim of this study was to compare the diagnostic accuracy between conventional forceps biopsy and endoscopic submucosal dissection (ESD).

Four hundred forty-four patients who finally undertook ESD in our hospital were enrolled from Jan 1, 2009 to Sep 1, 2015. We retrospectively assessed the characteristics of pathological results of CFB and ESD.

The concordance rate between CFB and ESD specimens was 68.92% (306/444). Men showed a lower concordance rate (63.61% vs 79.33%; *P* = 0.001) and concordance patients were younger (*P* = 0.048). In multivariate analysis, men significantly had a lower concordance rate (coefficient −0.730, *P* = 0.002) and a higher rate of pathological upgrade (coefficient −0.648, *P* = 0.015). Locations of CFB did not influence the concordance rate statistically.

The concordance rate was relatively high in our hospital. According to our analysis, old men plus gastric fundus or antrum of CFB were strongly suggested to perform ESD if precancerous lesions were found. And young women with low-grade intraepithelial neoplasia could select regular follow-up.

## 1 Introduction

Gastric cancer remains to be the third leading cause of cancer-related death worldwide.^[1]^ The early diagnosis of gastric cancer is still difficult. Unlike advanced-stage gastric cancer patients, patients with early-stage cancer and precancerous lesions usually have no symptoms. Once the onset of symptoms, it often indicates advanced-stage cancer. Therefore, the early diagnosis of gastric cancer is crucial to improve the survival rate. In some Asian countries, gastric cancer screening using endoscopy has been performed as an important method.^[2]^ Through the screening by endoscopy, we could find carcinoma in situ, precancerous lesions including low-grade intraepithelial neoplasia/dysplasia (LGIN) and high-grade intraepithelial neoplasia/dysplasia (HGIN)^[3]^ as soon as possible.

Conventional forceps biopsy (CFB) under endoscopy often selects locations of atrophy, erosion, ulcer, polyps. Once pathological diagnosis of carcinoma in situ, LGIN, HGIN by CFB, gastroenterologist usually advises patients to perform endoscopic submucosal dissection (ESD) to resect affected tissues completely and prevent further canceration. However, CFB does not represent the entire affected lesion because only a small portion of the lesion is sampled.^[4,5]^ Therefore, the CFB technique may underestimate gastric epithelial dysplasia lesions. Jeon et al^[6]^ reported that overall histological concordance rate between the endoscopic forceps biopsy and ESD specimens was 81.1% (107/132). Although ESD is minimally invasive compared with operation, there are still some potential complications including bleeding or perforation as well as time and cost of care without proven long-term benefits.^[7]^ Therefore, estimating the pathological diagnosis of CFB before ESD is necessary.

In this study, we retrospectively assessed the characteristics of pathological results of CFB and ESD in our hospital and calculated the concordance rate. Through analysis, we can conclude who is more suitable for conservative treatment and who is not.

## 2 Methods

### 2.1 Patients

From January 1, 2009 to September 1, 2015, we retrospectively enrolled 444 patients finally undertaking ESD in the First Affiliated Hospital, College of Medicine, Zhejiang University. Patients were included in the study according to the following criteria: informed consent was provided before ESD; older than the age of 18; conventional forceps biopsy was also performed before ESD. And patients were excluded according to the following criteria: patients had a clear history of gastric cancer; age <18-year old; patients had no pathological diagnosis of CFB; pathological diagnosis of CFB showed mild inflammation, ultrasound gastroscopy, or confocal laser endomicroscopy highly suspected gastrointestinal stromal tumors (GIST) or heterotopic pancreas, and ESD diagnosed GIST or heterotopic pancreas finally. This study was approved by Ethical Committee of College of Medicine, Zhejiang University.

In addition, characteristics of age, gender, degree of education, and areas of CFB were all selected.

### 2.2 Histological evaluation

The histological diagnosis was determined according to the World Health Organization classification^[8]^ and Gastric cancer diagnosis and treatment specification guidelines of Ministry of Health of the People's Republic of China^[9]^: the most important characteristics of LGIN are mild atypia of mucosal glands structure and cytology, dense cell nucleus, and nuclear fission; HGIN or carcinoma in situ is considered if severe atypia of mucosal glands structure and cytology, severe disorder of the gland cell arrangement and polarity, active nuclear fission, and focal necrosis are found; a diagnosis of adenocarcinoma is based on neoplasm with invasion.^[9]^

In our study, pathological types were divided into 4 categories: chronic inflammation change (CIC) including hyperplastic polyp; LGIN; HGIN; adenocarcinoma. All CFB pathological results of patients were compared with ESD (Table 1).

**Table 1:**
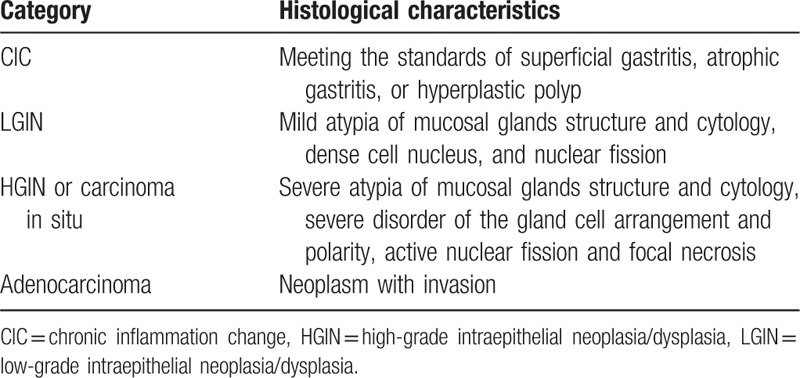
Histological categories and characteristics.

### 2.3 Statistical analysis

In our study, concordance rate of CFB and ESD was the main outcome which we wanted to know. According to different influence factors such as gender, age, locations of CFB, we analyzed which really influenced concordance rate.

Univariate analysis was performed using Student *t* test for the continuous variables such as age. And *χ*^2^ test was used to compare categorical variables such as gender, education, locations of CFB. In addition, pair-wise comparison among multicategorical variable groups was performed using *χ*^2^ test combined with Bonferroni correction.^[10]^

Multivariate analysis was performed using logistic regression and coefficients of variables for multivariate model were estimated. The statistical significance was defined as *P* <0.05. We used SPSS 21.0 (IBM, Chicago, IL) to perform the statistical analysis. Another associated data were calculated and plotted using GraphPad Prism 5 (Graph Pad, San Diego, CA).

## 3 Results

### 3.1 Baseline characteristics

The mean age of the enrolled patients was 60.21-year old (60.21 ± 10.06). Among them, man occupied 66.22% (294/444), while woman was 33.78 (150/444). In addition, degree of education of individuals lower than senior high school was 78.83% (350/444). According to the site of CFB, gastric antrum occupied 61.26% (272/444), gastric body occupied 10.59% (47/444), gastric angle occupied 17.79% (79/444), gastric fundus occupied 1.58% (7/444), and cardia occupied 8.78% (39/444).

### 3.2 Pathologic results and histological concordance rate between CFB and ESD specimens

**Figure 1:**
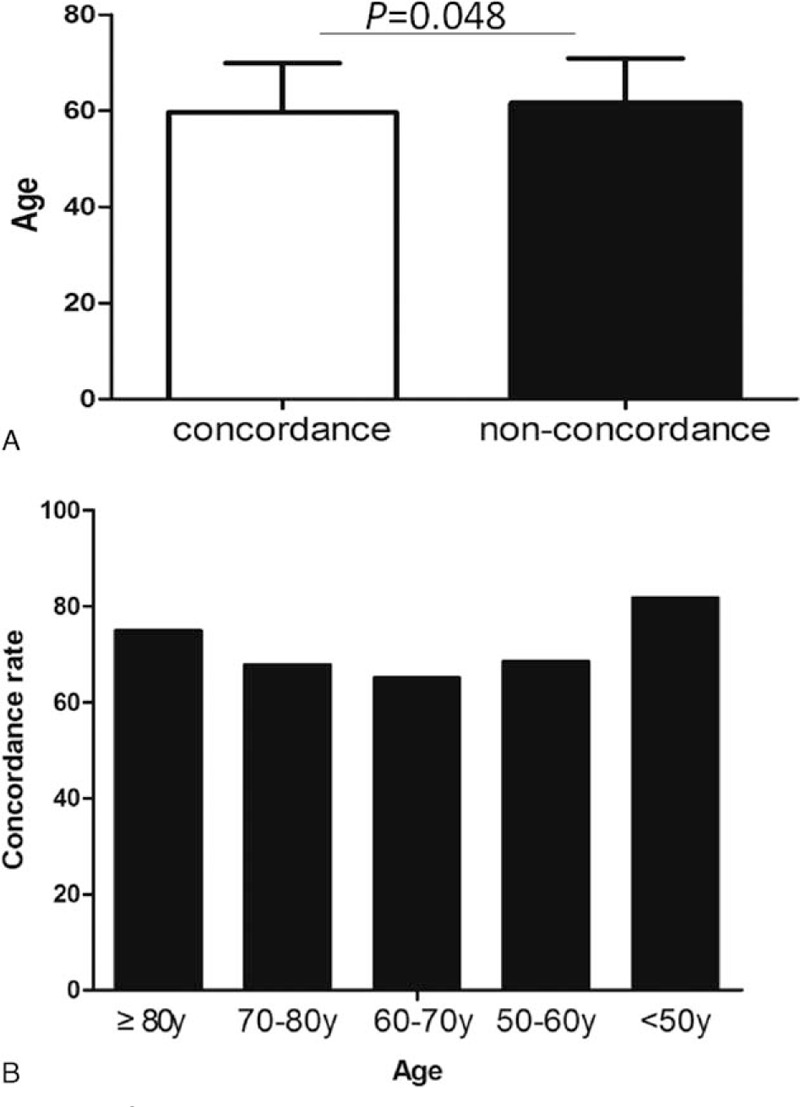
A, Comparison of ages between concordant and nonconcordant patients (69.58 ± 10.34 vs 61.62 ± 9.3; *P* = 0.048). B, Comparisons for every 10 years old of concordance rate, no statistical difference was found (*P* >0.05).

**Figure 2:**
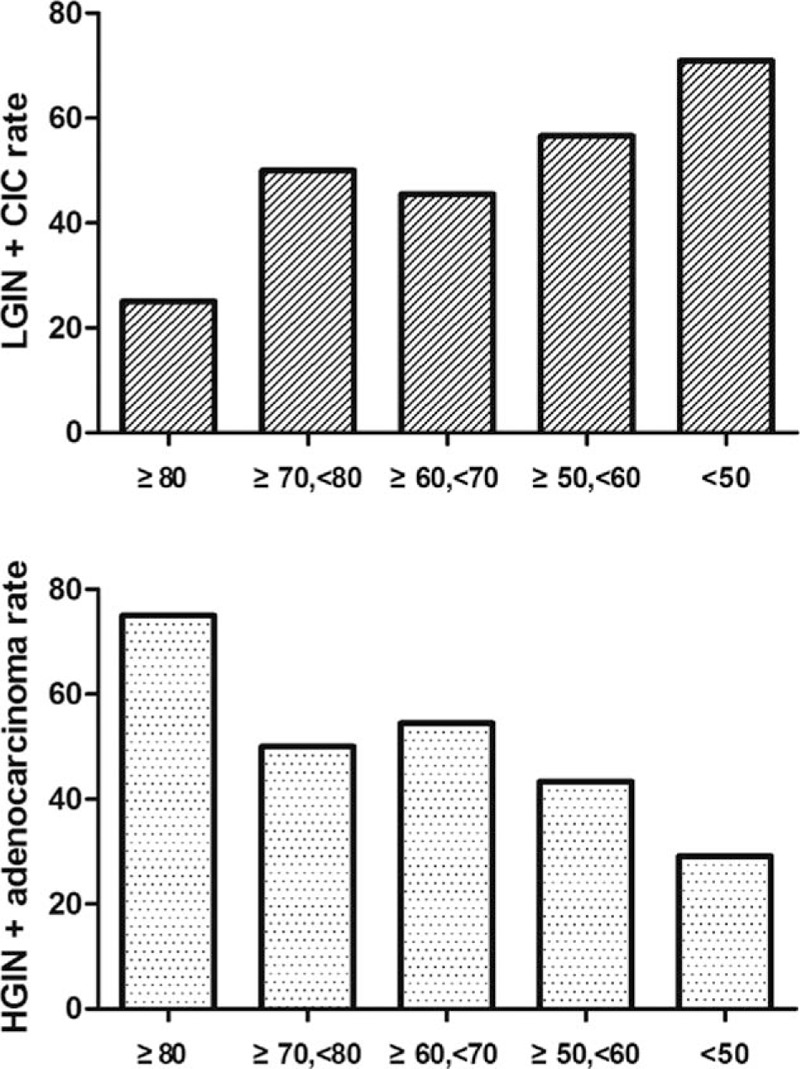
A relative increasing trend of HGIN-adenocarcinoma rate and a relative decreasing trend of LGIN-CIC rate can be observed with the increase of every 10-year old.

**Figure 3:**
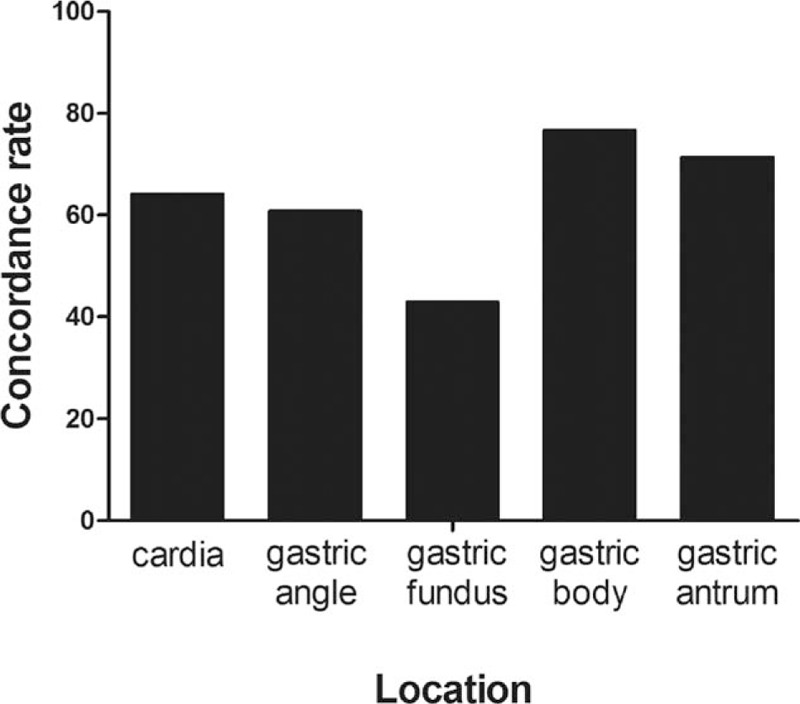
Comparisons for 5 gastric locations of CFB of concordance rate, no statistical difference was found.

**Figure 4:**
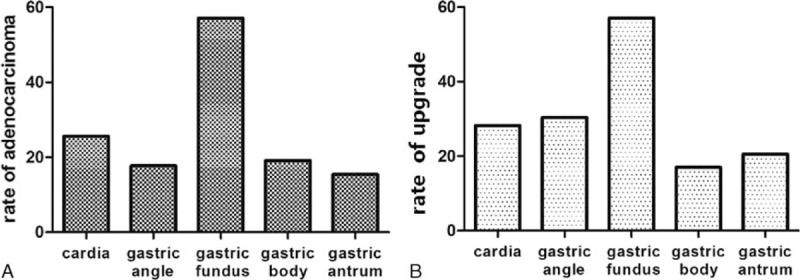
A, According to 5 gastric locations of CFB, the comparisons of rate of adenocarcinoma. B, According to 5 gastric locations of CFB, the comparisons of rate of pathological upgrade.

The pathologic results from CFB and ESD specimens are shown in Table 2. The overall pathologic concordance rate between the CFB and ESD specimens was 68.92% (306/444) among the enrolled patients. Among them, the concordance rate of LGIN was 77.21% (166/215), while HGIN was 50.56% (91/180) (*P* <0.001). In addition, we found that men had a lower concordance rate than women (187/294 vs 119/150; *P* = 0.001). The LGIN concordance rate reached up to 85.06% (74/87) of women. The patients of accordant specimens were younger than nonconcordance (69.58 ± 10.34 vs 61.62 ± 9.3; *P* = 0.048) (Fig. 1A). We divided patients into 5 classes every 10-year old. Patients older than 80-year and younger than 50 years had a relatively high concordance rate (75%, 81.82%), while patients (≥60, <70 years) had the lowest rate (Fig. 1B). But there was no statistical difference. Some information still can be observed. Although concordance rates were both high, patients ≥80 year had a higher rate (75%, 9/12) of HGIN and adenocarcinoma in final ESD pathology, while patients <50 had a higher rate (70.91%, 39/55) of LGIN and chronic inflammation change (Table 3). A relative increasing trend of HGIN-adenocarcinoma rate and a relative decreasing trend of LGIN-CIC rate can be also observed with the increase of age (Fig. 2). Moreover, there was no statistical difference between high education and low education (64/94 vs 242/250; *P* = 0.844). In 5 locations of CFB, gastric fundus showed the lowest concordance rate (42.86%, 3/7), while gastric body showed the highest (76.6%, 36/47) (*P* >0.05) (Fig. 3). Interestingly, gastric fundus had a highest rate of adenocarcinoma (57.14%, 4/7) (Fig. 4A) and a highest rate of upgrade of CFB pathological results (57.14%, 4/7) (Fig. 4B).

**Table 2:**
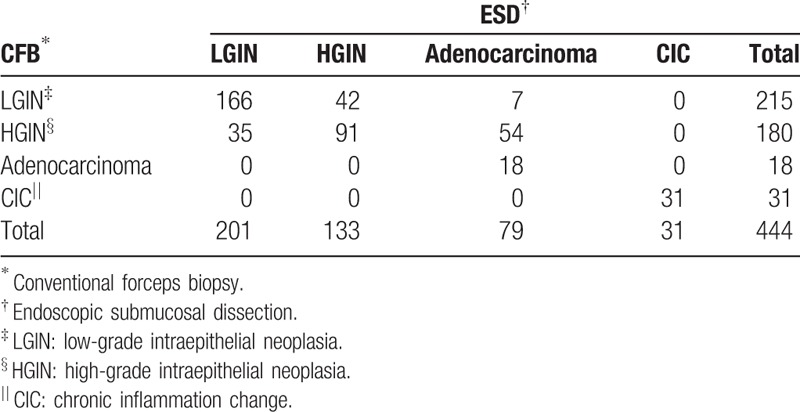
The pathologic results of CFB and ESD specimens.

**Table 3:**
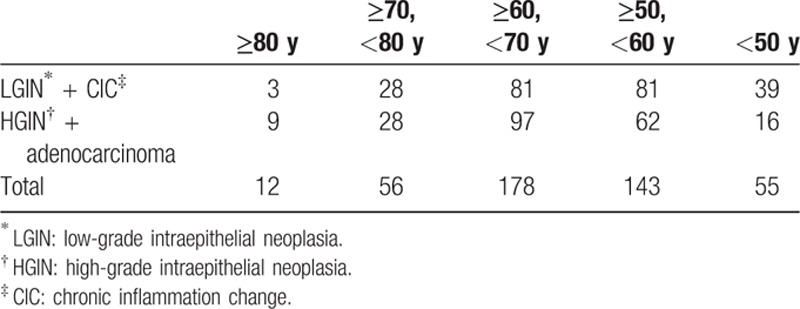
ESD characteristics of different age groups.

### 3.3 Multivariate analysis related to the histological concordance between CFB and ESD specimens

On multivariate analysis, gender still influenced the concordance rate as an independent factor (coefficient −0.730, *P* = 0.002). Men showed a decrease concordance rate. Age just showed a trend and there was no statistical significance (coefficient 0.019, *P* = 0.083). Moreover, other parameters such as education, location of CFB were not associated with pathological concordance (Table 4). In addition, men showed a higher rate of pathological upgrade (coefficient −0.648, *P* = 0.015) and gastric fundus had a trend of pathological upgrade (coefficient 0.048, *P* = 0.091) (Table 4).

**Table 4:**
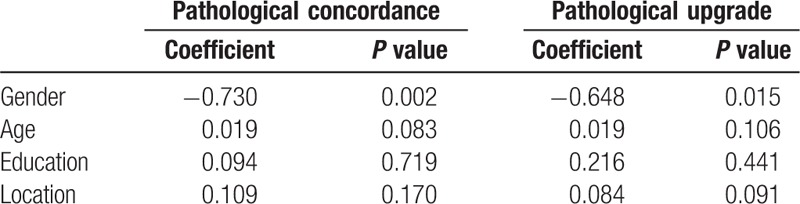
Multivariate analysis of pathological concordance and upgrade.

## 4 Discussion

In our study, through the retrospective study of 444 patients in our hospital, the concordance rate between CFB and ESD reached up to 68.92%. If we included patients whose CFB pathological results were HGIN while ESD were adenocarcinoma, the concordance rate would reach up to 81.08%. We could say that this result was satisfactory.

The concordance rate of LGIN in our study was 77.21%. For the treatment of LGIN, the Vienna classification recommends 2 options, local resection or follow-up.^[11,12]^ It seemed that follow-up could become a more suitable way. Previous prospective long-term follow-up study had indicated that the 5-year gastric cancer incidence in LGIN was 17%.^[13]^ However, the CFB may not represent the entire lesion of stomach, and this can result in underestimation of the possibility of coexisting HGIN or cancer.^[5]^ In addition, different pathologists may diagnose different results. Hull et al indicated that 45.2% of patients with LGIN who underwent ESD had discrepant diagnoses.^[14]^ In our study, it also happened. Pathological results of 49 patients (22.79%) upgraded. Won et al also suggested that ESD should be widely considered for large lesions or lesions containing a depressed region because of the closely related to hidden cancer or histologic progression.^[5,15]^ Further multilocations of CFB may improve the accuracy rate. Several molecular markers such as APC mutation and methylation of the p16 gene are related to the malignant transformation of gastric dysplasia.^[16,17]^ According to our study, young women without locations of gastric fundus could select routine endoscopic follow-up as a prior treatment. In addition, CFB just costs ¥ 100 to ¥ 200, while ESD needs ¥10,000 to ¥ 20,000. Obviously, the cost/benefit ratio of ESD treatment is lower.

Endoscopic resection is strongly recommended for HGIN, because of high possibility of evolving into adenocarcinoma.^[18,19]^ A study also reported that more than 80% of HGINs progress to adenocarcinomas.^[20]^ In our study, 54 patients (30%) showed adenocarcinomas under ESD while HGINs under CFB. It was a high ratio. By contrast, only 3.26% showed adenocarcinomas under ESD while LGINs under CFB. Therefore, HGIN is a strong indication to perform ESD. In addition, we also observed an interesting phenomenon. Thirty-five patients (19.44%) showed LGINs under ESD while HGINs under CFB. Degradation of pathological diagnosis from ESD to CFB appeared. Possible causes may lead to this discrepancy: because of geographic variety of histology, spot or focal lesions of HGIN, lesions were removed under CFB. Kin et al^[21]^ reported that 20 cases found nonneoplastic pathology results under ESD/EMR while LGINs/HGINs/cancers were found under CFB. In China, cases of spot cancers had been reported many times^[22]^; chronological difference between the time of CFB and ESD, as drugs application, some lesions appeared to reverse of pathology; a handful of cases may occur at the different locations of CFB and ESD, but it did not happen in our study. According to our analysis, old men plus gastric fundus of CFB were strongly suggested to perform ESD if precancerous lesions were found because of high disconcordance rate.

In our study, we found gastric antrum was still the major location of precancerous lesions and cancers. But we did not ignore the high proportion of discordance rate and upgrade of pathological result in gastric fundus. The question arose, how could we improve detection rate and concordance rate to reduce unnecessary surgery. Multilocation of CFB is a rational way to improve concordance rate^[6]^ especially to old men, because of the rising tread with age of disconcordance rate. However, the diagnostic accuracy did not significantly increased by the use of jumbo forceps biopsy.^[6]^ In addition, other endoscopic technology such as confocal laser endomicroscopy, magnifying endoscopy, chromoendoscopy, narrow-band imaging, autofluorescence imaging may help clinicians improve the diagnostic rate. Some immunohistochemical and tumor markers such as CA72-4 could offer auxiliarily diagnostic basis. Moreover, considering the confusing differentiation between regenerative atypia and LGIN, pathological reevaluation of CFB is also necessary.^[23,24]^

Although we obtained the ideal result, the study also had several limitations. First, the main limitation was potential selective bias in our retrospective study. Patients with high education and high income may have more opportunity to perform periodic physical examination and ESD. In addition, we excluded patients diagnosed as heterotopic pancreas and GIST by confocal laser endomicroscopy or endoscopic ultrasonography, then ESD further diagnosed. The reason why we excluded these patients was that patients’ pathological results of CFB showed mild-to-moderate inflammation and other endoscope techniques had more diagnostic value. Second, because of the workload, no more pathologists verified pathological results repeatedly. So it may improve the disconcordance rate. Third, we did not have standardized criteria regarding the next treatment. Every clinician has his own judgment standard for LGIN about recommending ESD or follow-up, which may also manufacture bias. Therefore, it is necessary to conduct a further large-scale prospective study to overcome these limitations.

## 5 Conclusions

In summary, according to our data, we found that men had a lower concordance rate and observed decreasing tendency of concordance rate as the growth of the age. Gastric antrum was the major location of precancerous lesions and cancers, while gastric fundus had the high proportion of discordance rate and upgrade of pathological result. Therefore, old men plus gastric fundus or antrum of CFB were strongly suggested to perform ESD if precancerous lesions were found. And young women with LGIN could select regular follow-up. A further large-scale prospective study is also needed to provide more evidence.
